# Baby Week

**Published:** 1917-06-16

**Authors:** 


### BABY WEEK.

It is by no means easy to stir the British public. Matters of the greatest importance may present themselves, but it often happens that the British mind is hardly ruffled, and that which might have been expected to give rise to some energetic action is passed over almost without notice. Yet when the British public is really aroused, when the importance of a matter is fully appreciated, nothing can exceed the purposeful energy with which the matter is taken up at least for a time, and with the best intentions many things done which may prove to be not merely useless, but even actively harmful. So has it been with Baby Week. At last after many years the baby is being appreciated as he deserves; the need for babies is apparent to all; gone are the times when advertisements were inserted for married couples "without encumbrances." The awful slaughter in the war, the loss of the youngest, the bravest, and the best of our men has penetrated the most pachydermatous minds, and all feel that if we are not to fall behind in the world race we must see that there is no unnecessary loss of the babies that are born. To accentuate this recognition of the need to prevent the waste of infant life a "Baby Week" has been arranged. That there is a lamentable ignorance amongst parents of the way in which to rear a child there can be no doubt; the very heavy mortality in young children, and especially in the first year of life, is known to all, and for the most part this excessive mortality is attributable to lack of care in the feeding and nurture of the child. Necessarily to a certain extent the poverty of the parents is a contributory factor, but its influence will not be great, for far more often the food given is generous in quantity, but is unfortunately absolutely unsuitable in kind. All who have worked in children's clinics know the lamentable lack of knowledge of how to bring up a child amongst the mothers of the working classes, and there can be no doubt that with careful instruction many mothers may learn much that will enable them to better provide for and safeguard their children. In only too many instances the baby at his very birth is not in a condition to be easily reared; he enters life's race heavily handicapped by heredity, and even more by the ill-health of his mother during her pregnancy. How much may be done to improve hereditary tendencies it is hard to say, but it is beginning to be recognised that if the new-born child is to have the best chance to become a healthy member of the community, one of the most important factors is the adequate care of the mother before the child is born.

All these points and many others are to be considered and discussed at the forthcoming congresses to be held in "Baby Week." Elaborate arrangements are being made to hold exhibitions of a multitude of things and matters pertaining to the baby, before and after birth, and there will be issued a full account of all that is to be done. We cordially welcome the whole movement; it was sadly needed, and is in the right direction, but care must be taken to provide against zeal without knowledge, and to eliminate everything which would in no way advance the cause which we all have at heart. What a baby really requires is very simple. That his food should be pure and good is essential, and if it can be obtained there is nothing better than the milk of a healthy mother. Of fads as to his care, clothing, and surroundings there are many, but to succeed, those responsible must secure their suppression.

The excitement of "Baby Week" will die away; many of the plans and arrangements will be forgotten, but the hardest task of all will remain. That will be to keep up the interest of the public and to guarantee the continuous better care and upbringing of British babies for the future.

